# Single-Camera-Based Bridge Structural Displacement Measurement with Traffic Counting

**DOI:** 10.3390/s21134517

**Published:** 2021-07-01

**Authors:** Zulhaj Aliansyah, Kohei Shimasaki, Taku Senoo, Idaku Ishii, Shuji Umemoto

**Affiliations:** 1 Graduate School of Engineering, Hiroshima University, 1-4-1 Kagamiyama, Higashi-hiroshima, Hiroshima 739-8527, Japan; aliansya@robotics.hiroshima-u.ac.jp; 2 Digital Monozukuri (Manufacturing) Education Research Center, 1-3-2 Kagamiyama, Higashi-hiroshima, Hiroshima 739-8511, Japan; simasaki@hiroshima-u.ac.jp; 3 Graduate School of Advanced Science and Engineering, Hiroshima University, 1-4-1 Kagamiyama, Higashi-hiroshima, Hiroshima 739-8527, Japan; taku-senoo@hiroshima-u.ac.jp; 4 Keisoku Research Consultant Co., 1-665-1 Fukuda, Higashi-ku, Hiroshima, Hiroshima 732-0029, Japan; umemoto@krcnet.co.jp

**Keywords:** bridge vibration, displacement distribution measurement, digital image correlation, structural health monitoring, traffic counting, vehicle classification

## Abstract

Vision-based structural displacement methods allow convenient monitoring of civil structures such as bridges, though they are often limited due to the small number of measurement points, constrained spatial resolution, and inability to identify the acting forces of the measured displacement. To increase the number of measurement points in vision-based bridge displacement measurement, this study introduces a front-view tandem marker motion capture system with side-view traffic counting to identify the force-inducing passing vehicles on the bridge’s deck. The proposed system was able to measure structural displacement at submillimeter resolution on eight measurement points at once at a distance of 40.8–64.2 m from a front-view camera. The traffic counting system with a side-view camera recorded the passing vehicles from two opposing lanes. We conducted a 35-min experiment for a 25 m-span steel road bridge with hundreds of cars passing over it and confirmed dynamic displacement distributions with amplitudes of several millimeters when large vehicles passed.

## 1. Introduction

Bridges have served a vital part in the transportation system by allowing road traffic and other loads to traverse over physical obstructions caused by topological and structural conditions, e.g., rivers, valleys, railways, elevated roads. A bridge’s inability to serve its purpose may result in a longer travel route or an isolated area removed from the transport network. Bridge failures often result in casualties and large-scale monetary loss; thus, such failures need to be prevented. A bridge is constantly subject to damages from environmental factors or inherent usage loads during its service life. The damages to a bridge can be categorized into three kinds of effects [1]:

- Primary effects—related to the bridge’s material, construction type, shape, and design considerations in relation to the bridge’s expected load and its dead weight under static condition.
- Secondary effects—identified from the time-bound dynamic structural response excited from forcing actions due to vehicular loads, wind, quake, or thermal expansion/shrinkage, which are often nonlinear and inelastic.
- Tertiary effects—not directly related to static nor dynamic properties of the bridge, which include environmental factors such as vegetation overgrowth, rust on metal reinforcing members, paint decontamination, water ponding, deck spalling, erosion, silting, etc. [2].

Although these three effects are in interplay with one another, addressing them is often done separately. Primary effects should have been well-addressed in the design phase and carried well throughout the construction. Secondary effects are to be evaluated using finite-element simulation or direct measurements on the structure under a defined load. Tertiary effects are usually assessed by routine visual qualitative inspection on the structure.

Condition assessment and maintenance need to be performed to maintain the safety and durability of the bridge, especially to address the second and third effects of damage. Early damage detection from structural health monitoring (SHM) facilitates more economical management and maintenance of modern infrastructure in the long run, given the bridge’s longer expected service life, relative ease to address the early stage of the damage, and prevention of damage accumulation [3]. A convenient method for structural health monitoring should be realized to address the vast number of bridges in lieu of a sophisticated arrangement of instruments [4,5], and ease of installation and flexibility of deployment in the field should be taken into account to minimize social economic losses with traffic regulation in addition to the resolution and accuracy of the measurements.

In this study, we introduce a single-camera-based deflection distribution measurement method with subpixel digital image correlation (DIC) analysis for a dozens-of-meters-span road bridge; this is extended from the tandem-marker-based motion capture method [6] that allows measurement of structural dynamic displacement from multiple tandem-distributed points at once without decreasing measurement accuracy by installing a front-view camera positioned with a small angular delineation to the bridge’s axis. It also enables easy-installation bridge monitoring without traffic regulation. A traffic counting system with a side-view camera was additionally installed for identifying the dynamics response of the bridge specific to the passing vehicular traffic, and the relationship between the sizes of passing vehicles and their responses were quantified in bridge deflection measurement.

## 2. Related Works

### 2.1. SHM with Displacement Measurement

Bridges experience elastic vertical, lateral, and rotational displacement from the service load combinations, and such load displacement may cause surface deterioration and local cracking, impairing serviceability and durability [7]. Continuously unattended excessive displacement may result in the accumulation of structural damages in the bridge, reflected in the loss of stiffness of weakened parts of the bridge: bearings, joints, integral abutments, and piers. To restrict excessive bridge deformations and vibrations, the displacement limit is determined by linking with human psychological perceptions on structural safety due to the occurring structural responses [8], as well as inspecting the appearance of sagging in the bridge girders.

Structural damages are quantified with metrics derived from displacement, such as increase of the frequency response in a specific frequency band, a shift of the bridge’s modal frequency peaks toward a lower value, a discontinuity in the mode shapes, lower damping coefficient, and other anomalies [9,10].

Frequency-domain analyses are usually employed on the extracted vibration signals to evaluate the structural health of bridges [11,12]. The frequency response from a bridge’s natural frequency and traffic load often resides at separate frequency bands [13]; thus, identifying the frequency response specific to the structure would be possible. Statistics of abnormal modal frequencies can infer structural damages [14]. Although the natural frequencies may exhibit variance between multiple methods, measurement points, and ambient loading conditions [11,13], frequency-domain analysis gives reasonable estimates from the extracted vibration signal. As the structural damage is often localized within a particular member or joint, simultaneous measurement on multiple points on the bridge is also important to pinpoint the damage’s approximate location.

### 2.2. Drive-by Bridge Vibration Measurement

Vibration-based SHM refers to in-field nondestructive displacement sensing and analysis of a structure in the time, frequency, or modal domains. Changes and irregularities in these domains may indicate damage or degradation. Damage to a bridge may lead to some stiffness loss and, consequently, change its dynamic properties [15,16]. Loss of stiffness could be reflected from a displacement larger than the design value or a drop in the vibration frequency response given a specified loading condition. In practice, the specific loading condition is performed either using heavily loaded trucks parked in specified points along the span of the bridge [17]; large, controlled excitation devices attached to a specific vehicle [18]; or, more recently, with drive-by methods utilizing ambient excitation forces from the usual traffic [19,20].

Testing the vibration of a bridge from vehicular traffic is intuitive, as the bridge’s service will still operate as usual and can potentially be conducted continuously in real-time. However, in practice, the interaction between vehicles and the bridge will vary due to factors related to the bridge construction (length, dead mass, deck surface roughness, thermal stress on the members) [14], the passing vehicles (axle spread, weight, speed, suspension) [21], or the superposition of multiple vibration sources [22].

The impact of vehicle load on bridge vibration varies by the weight on each axle, the distances between axles, and the number of axles. Vehicles need to be configured in such a way that the excitation force will result in dynamic displacement, which is discernible from ambient vibration from the bridge’s dead weight and environment [23]; thus, heavy vehicles are often used for various tests on a bridge instead of common car traffic. Vehicle speed and bridge deck roughness also affect the vibration profile of the bridge. At higher speeds, vehicle vibration dominates the observed vibration signal, hiding the bridge vibration itself, and the vehicle speed needs to be relatively low to obtain good separation between bridge and vehicle vibration frequency, as previously reported: 18 km/h [23], 36 km/h [24], and 40 km/h [19].

### 2.3. DIC Analysis

DIC is a method to infer subpixel deformation between two images using the correlation of defined facet [25] from the object’s normal surface, nonperiodic speckled pattern, or target marker apparatus. DIC evaluates various criteria from the facet in a single 2D-plane [26], such as cross-correlation, sum of absolute difference, and the squared sum of difference. The adjacent frames can also be evaluated in either the forward or backward temporal direction [27]. DIC can be expanded for multiplane measurement using stereo camera heads, beam-splitter prisms or mirrors, or wavelength separation of the exposures [28]. Measurement in the 3D-plane can be achieved with a digital projection of the facet or oblique arrangement between the camera and object with known distances [29]. The latter is more common for displacement measurement of structures.

Various DIC applications have been demonstrated on a model bridge to measure displacement, strain, and modal responses [11,30,31,32]. DIC shows comparable results to conventional methods with accelerometers, laser vibrometers, or finite element simulation [11,33], despite its limitations regarding changes in facet appearance [31], image distortion and out-of-plane displacement or rotation [34], limited sampling rate [35], and stray frequency responses from sensor aliasing or ambient vibration [33].

DIC has been implemented on actual bridges in several studies addressing the issues of real-time performance, image degradation from optical turbulence, and subpixel accuracy [36,37,38]. Such implementation has yet to consider moving traffic and address the inaccuracy due to imprecise synchronization of multiple cameras [39]. There is a trade-off between the field of view and spatial resolution due to finite sensor size and focal length. Usage of a pan-tilt mechanism allows for a wider field of view without resorting to a lower pixelwise spatial resolution [40], but the setup may introduce unwanted vibration and image distortion from the varying object distances. A higher spatial resolution could be achieved by applying a complex filter to a downsampled subset of the image [30,36].

### 2.4. Video-Based Traffic Counting

A traffic counting system detects individual passing vehicle and infers their properties: time of passage, travel direction, length, and estimated velocity. These information can be used for various purposes: congestion detection, automated tolling, road design, speed limit enforcement, etc. The automated traffic counting system has been implemented with various detectors, e.g., induction loop, speed-trap laser, and surveillance camera. The video-based traffic counting system is characteristically more robust with a long MTBF (mean time between failures) compared with other detectors [41].

Video-based traffic detection of passing vehicles has been implemented with various techniques: MoG (mixed of Gaussian) [42] and frame-history background subtraction [43] can filter out the nonstatic multi-modal background (e.g., shaking leaves, swaying branches, shadows), but both can still result in the false detection of slow-moving or stopping vehicles, and overlapping vehicles (either from a single lane or adjacent lanes). Color-histogram clustering addresses the issue with overlapping vehicles, but, in turn, exhibits inaccuracy due to sudden changes in vehicle velocity, illumination changes, and color composition variation [44].

The image segmentation method works to detect the presence of the vehicles by subtracting each captured frame with a background image with no vehicle present. The rolling average threshold for image segmentation proved to have good accuracy in various weather and lighting conditions [45], with detection lines facilitating detection in multilane settings with heterogeneous traffic [46]. This proves that a simple and adaptable image processing routine will suffice if detection thresholds are adjusted properly.

Classification of the detected vehicles has been conducted to infer their properties. SVM (support vector machine) classification is often inaccurate [42], and increased accuracy comes with the added cost of computational complexity [47]. The CNN (convolutional neural network) method promises better accuracy [42] but requires prior supervised learning for various different vehicle appearances, camera orientations, and lighting and weather conditions [48,49,50], and generally performs slower compared to image-processing-based methods. Thus, context-specific criteria should be applied to extract meaningful information from the passing vehicles to obtain better execution speed with acceptable accuracy.

## 3. Tandem Marker Motion Capture with Side-View Monitoring

### 3.1. Single-Camera-Based Bridge Displacement Measurement

Integration of structural health monitoring on a bridge and traffic identification allows the measurement of structural dynamic response from traffic load to assess the structural integrity of the bridge. Several studies have been carried out by integrating an array of sensors (strain gauge, accelerometer, weigh-in-motion plate) and traffic monitoring video cameras [51]. Video-based methods have also been applied in place of the sensors to facilitate noncontact measurement of the bridge vibration under live load, measuring the deflection of a portion of the structural members, e.g., girders [52] and cable stays [53]. Although such systems are generally comparable to finite simulation [54] and able to identify simulated damages [55], they are still constrained due to the finite number of measurement points, the requirement of synchronization between cameras [53], and the lack of generated data from the traffic counting camera—especially in a real-time setting [56].

The suboptimal common placement of both displacement measurement (perpendicular to the bridge’s axis) and traffic counting cameras (high vantage point) resulted in a set of issues: limited measurement field and the requirement of separate calibration/alignment for each displacement measurement camera [52]; further, the difficulty to estimate the passing vehicle’s details (size, type, speed) and susceptibility to errors are related to frontal occlusion for the traffic counting camera [42,57,58].

Tandem arrangement of the optical markers along the span of a bridge with the frontal placement of the displacement measurement camera has been found to facilitate simultaneous capturing of multiple displacement measurement points from a single camera viewpoint in its depth of field, requiring no synchronization module for multiple-point measurement [6]. This could enable a simpler method of whole-span bridge displacement observation. The placement of traffic counting camera perpendicular to the bridge axis could also allow a more reliable estimate of the passing vehicles’ dimensions.

**Figure 1:**
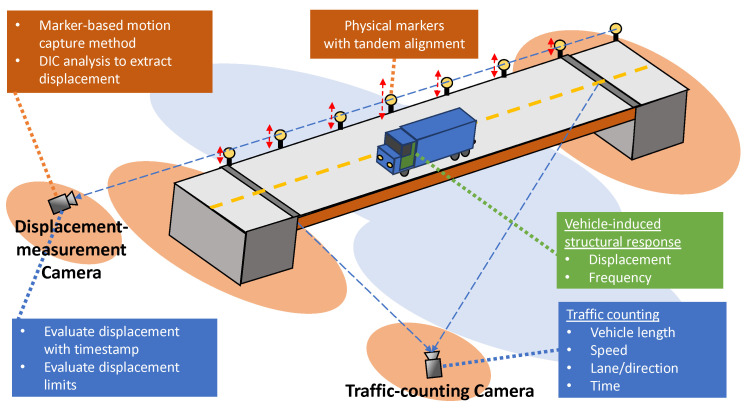
Framework of the SHM method for traffic-induced dynamic displacement measurement using marker-based motion capture and traffic counting cameras. The method incorporates a frontal-positioned camera to measure displacement from multiple points simultaneously and a side-positioned traffic counting camera that allows more-accurate capturing of passing vehicles. DIC and traffic counting algorithms are concurrently implemented to measure the displacement and frequency response of the traffic-induced vibration.

This study aims to identify the structural dynamic response of an actual bridge from various live traversing vehicular traffic, using a combination of the single-camera motion capture DIC technique and traffic counting system. The dynamic displacement signals from DIC analysis will be cross-referenced against traffic information using a synchronized timestamp. Figure 1 illustrates the general framework of the system.

### 3.2. Field Experiment

The field experiment was conducted on Hinotsume Bridge (34${}^{\circ }$21${}^{\prime }$27.2${}^{\prime \prime }$ N 132${}^{\circ }$43${}^{\prime }$17.4${}^{\prime \prime }$ E). The structure incorporates two steel plate girder spans with a concrete deck spanning in the east–west direction, which allows both vehicles and pedestrians to cross. Each span is approximately 25 m-long with a support pier in the center connecting the two spans. The two-lane roadway on top of the structure is 8.4 m-wide with adjacent sidewalks 2 m-wide on both sides. Guardrails are featured on the edges of the bridge and also between the sidewalks and the roadway. The speed limit in the vicinity is 50 km/h.

**Figure 2:**
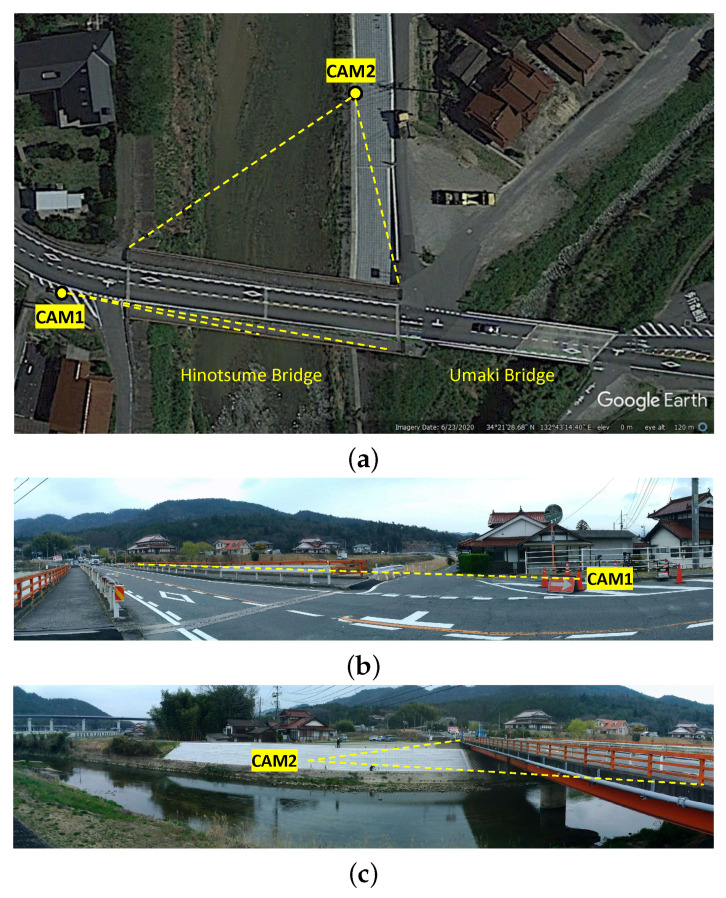
Field of view from the top view (**a**) of the displacement measurement camera (CAM1) (**b**) and traffic counting camera (CAM2) (**c**). The displacement measurement camera was positioned 64.2 m westward from the furthest marker installed on Hinotsume Bridge. The traffic counting camera was placed northward by the riverbank away from the bridge’s side.

The displacement measurement camera was installed at the shoulder of the road to the west of Hinotsume Bridge facing eastward. With its depth of field mostly parallel to the bridge’s axis, this allows simultaneous capturing of tandem-layout markers installed on the bridge without occlusion. The traffic counting camera was installed north of the bridge by the riverbank to capture the whole span of the bridge from the perpendicular direction, as shown in Figure 2. The perpendicular placement to the traffic direction may allow more robust detection and length estimation of the individual passing vehicle.

**Figure 3:**
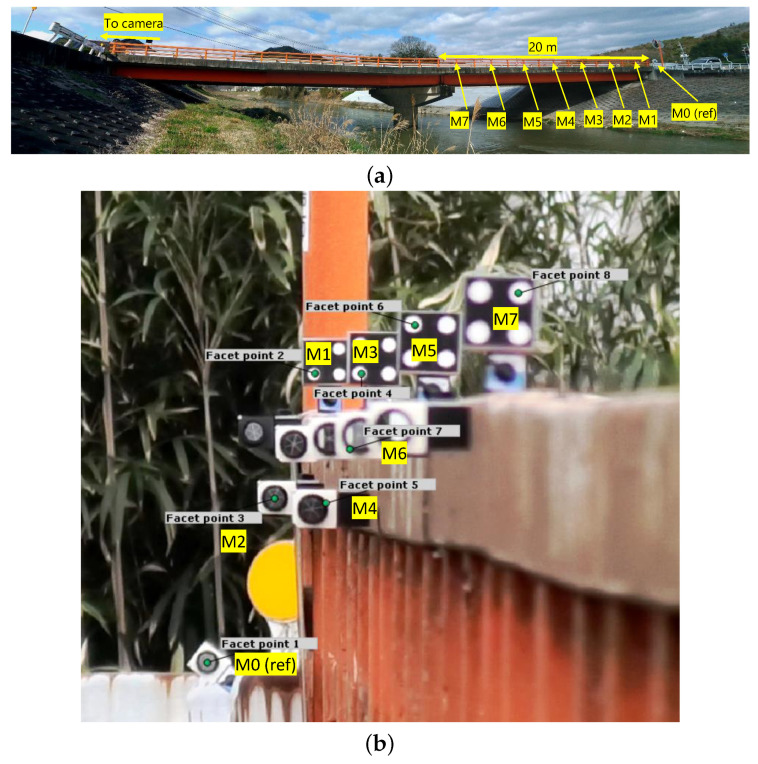
Markers’ placement on the bridge’s outer edge guardrail (**a**) and the cropped field of view from the displacement measurement camera with the markers’ facet assignment for DIC analysis (**b**).

Cameras for the experiment are Panasonic HC-WZX2M at 1920 × 1080 px resolution. Frame rate was 60 fps with 1 ms-exposure, short enough to eliminate motion blur while still bright enough to detect the facets for DIC analysis reliably. Focal length of the displacement measurement camera was set to 98.9 mm with F/16 aperture to obtain as much depth of field and the sharpest focus possible at maximum magnification. The duration of the experiment was 35.4 min during afternoon hours (15:49 to 16:25 JST). The daylight during the experiment provided enough light for proper exposure even at its narrowest aperture and no vignetting was observed in the captured frame of the displacement measurement camera, as shown in Figure 3b.

The markers were installed on the edge guardrail of the eastern span approximately 3.9 m apart from each other using a magnet holder stand (MiSUMi-VONA MNMGBSHLD, Japan), as shown in Figure 3a. Markers’ displacement was evaluated relative to the fiducial reference marker (M0), which was assumed to be static, installed on the abutment to the east of the bridge isolated from the deck, providing a mechanism to cancel camera head vibration. The movement of all other markers is evaluated relative to the movement of M0, as described in Equation (1). The markers were installed in a pattern such that all the markers could be captured without occlusion from one another at different distances from the camera, as shown in Figure 3b.

### 3.3. Implemented Algorithm

#### 3.3.1. Tandem-Marker-Motion Capture from Frontal Camera

**Figure 4:**
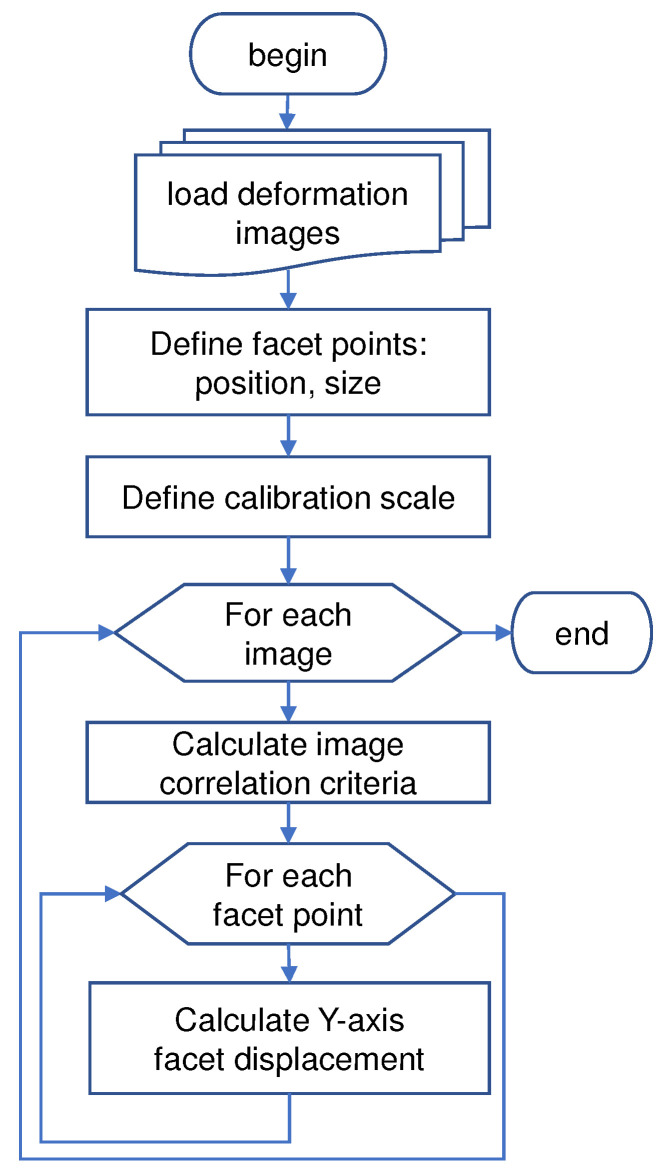
General workflow of DIC displacement measurement from multiple facets in a single plane. The deformation images were cropped beforehand to reduce the computational time of the DIC process. The images would be discarded from DIC analysis if occlusion occurs between the camera and markers due to traffic coming from the sides. Displacement of each facet is estimated based on the position of the region of the image with the highest correlation criteria across multiple frames.

Displacement measurement was performed using a commercial DIC solution (GOM Correlate 2019 Hotfix6 rev. 125216, GOM GmbH, Braunschweig, Germany), which features point-based and full-field displacements measurement. Only displacement in the vertical direction was evaluated in the analysis to emphasize the effect of vehicle loading on the bridge. Figure 4 explains the general workflow for the DIC displacement measurement process.

**Figure 5:**
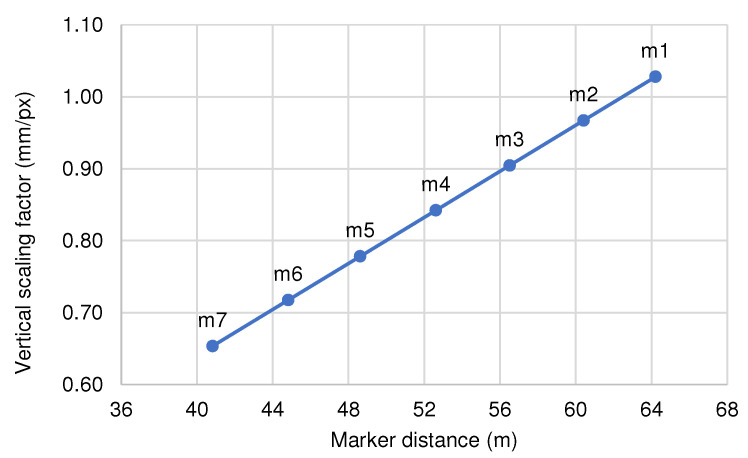
Scaling factor for each marker at different distances defines the ratio between actual physical displacement and perceived pixelwise movement in the captured image. The value was calibrated from the known object size of the printed marker (M1).

As the facets’ vertical displacement was evaluated from only a single axial plane, the calculated displacement of all markers was calibrated from the known object size of the printed pattern marker (60 mm) of marker M1. Displacement of each marker would need to be rescaled separately based on its respective distance to the camera. The approximate scaling factor for each marker is evaluated from the ratio of its respective distance to the distance of marker M1 from the camera, as specified in Figure 5, assuming no focal distortion from the displacement measurement camera throughout its field of view and no out-of-plane movement of the markers. Thus, the displacement for marker number *n* at time *t* is given as

(1) $$S_{n,t}=\frac{SF_{n}}{SF_{1}}\left(P_{n,t}-P_{0,t}\right),$$

where *P* is the captured pixelwise movement, *SF* is scaling factor for each marker, and *S* is the measured displacement.

Given that the displacement limit of a bridge is often defined using static displacement and the loading scenario during the experiment is entirely dynamic, single-point time-series static displacement from the structure is approximated using differential displacement. Differential displacement evaluates single-point displacement between the center of the span and abutment in the time domain, given as

(2) $$D_{t}=S_{4,t}-S_{7,t},$$

where *S* is the measured displacement of each marker at time *t*.

Differential displacement was calculated to compensate with the changing measurement baseline due to temperature, nonsteady traffic flow, and facet detection drift from the DIC process. Intermittent traffic jam and temperature changes have been shown to exhibit drift in the structural responses [59]. Butterworth band-pass filter (10th order, 1 Hz to 1.2 Hz band) was then applied on the differential displacement signal to suppress the vibration irregularities from the reverb vibration, vehicle suspension, road roughness, or superposition of vibration from other vehicles, and to better represent the first-mode vibration. Such signal smoothing has been proved to better extract static component of the bridge response [60].

Displacement from all markers shall be evaluated not to exceed the displacement limit set in the design to ensure structural safety. The displacement limit defined by AASHTO (American Association of State Highway and Transportation Officials) will be used for the evaluation. The limit considers the varying material, construction type, designated load, and perceived psychological safety of the structure [61], summarized in Table 1. Displacement over the specified limit indicates structural deficiency, although such deficiency may not lead straight to a failure and the bridge may subsequently still be able to serve typical loads within the limit. Nonetheless, repair or retrofit will be needed to ensure structural safety in the long run to avoid accumulation of damage.

Frequency-domain analysis is conducted to measure load-specific structural response from the bridge. The frequency spectrogram was evaluated using Fast Fourier Transform (FFT) from the measured displacement of M4 with a 128-samples bin at 33 ms step. The spectrogram refers to the amplitude of each frequency band as it varies with time. Only the lower-half of the frequency response band is presented for the analysis.

#### 3.3.2. Traffic Counting from Side-View Camera

There was persistent occlusion from the guard rails captured by the traffic counting camera which made capturing the vehicles’ full shape impractical. This may pose a challenge to neural-network-based object detection. Hence, in the experiment, vehicle detection was performed using basic image segmentation to reduce complexity and increase the traffic counting system’s overall performance. The traffic counting system was implemented on a laptop computer (HP Omen 15-dc1xxx, Intel i7-8750H, 32 GB RAM, python 3.8.6) and performed at 80 fps, citing the possible real-time application for the video stream captured from the camera at 60 fps.

**Figure 6:**
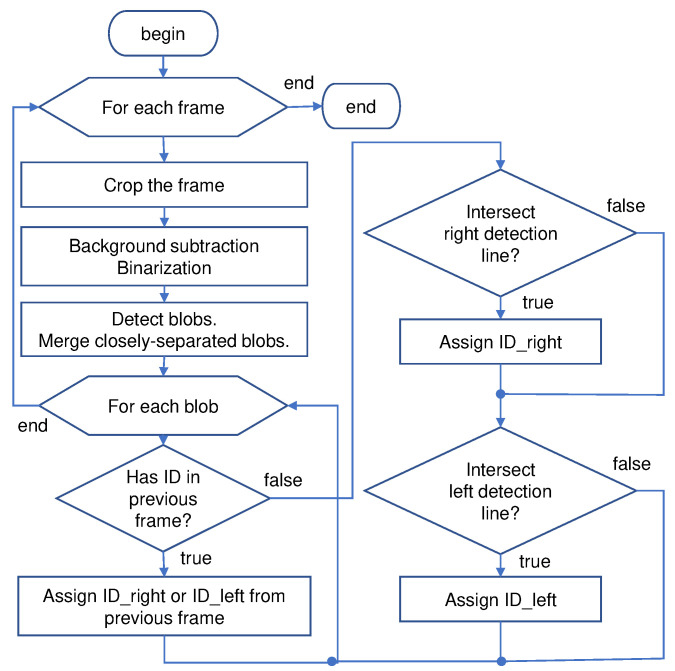
Flowchart for the traffic detection algorithm. Closely spaced blobs within 10 px apart are merged to account for the occlusion from the guardrail. A unique ID is assigned to a sizable blob (60 px-wide minimum) based on its initial position when crossing either of the detection lines. A single-vehicle blob can be assigned with two IDs (right-going <100, left-going >100) to handle momentary occlusion between left-going and right-going vehicles. The final ID is selected based on whether the ID value matches the movement direction.

**Figure 7:**
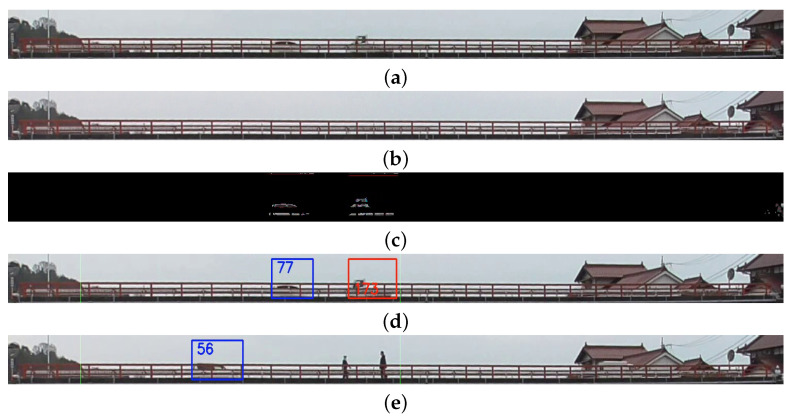
Captured frames from the video stream of traffic counting camera, cropped within the region of interest covering the bridge’s deck (**a**). The background image for the image segmentation process is captured when no vehicle is present on top of the bridge, updated with a 10-min interval (**b**). Detected vehicle blob after background subtraction (**c**), the current background image is subtracted from each frame to isolate only the moving objects. Detection frame with a unique ID assigned to the detected vehicles’ blob (**d**). The blob selection was able to filter out nonvehicular objects such as a walking pedestrian from vehicle detection based on minimum blob width (**e**).

Figure 6 explains the workflow of the image-processing routines for the traffic counting system. A random ID is assigned to the blob wider than 60 pixels, which is maintained throughout the pass’ duration between two defined line segments. Smaller blobs are regarded as nonvehicular traffic (pedestrian, motorcycle, etc.) or image noise from the environment. Figure 7 shows the images being handled from the traffic counting camera: captured image from the traffic counting camera, intermediate images in the image segmentation process, and the resulting image with tagged vehicles.

**Figure 8:**
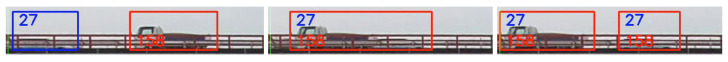
Image sequence of lane overlap between right-going vehicle (ID 27) and left-going vehicle (ID 158) before, during, and after the lane overlap. Traffic count data during the duration of lane overlap are discarded from the evaluation to ensure more representative data of respective vehicles.

Lane overlap occurs when two vehicles are coming from different directions and the right-going vehicle is occluded behind the left-going vehicle. At the first frame of such lane overlap, the ID from the opposing vehicle is doubly assigned to each other. This ensures that a specific ID is retained for a vehicle throughout its pass duration despite momentary lane overlap with other vehicle(s), as shown in Figure 8. By the end of the pass, the ID of each vehicle is checked to match with its travel direction, assuming that no vehicle is going in the reverse direction. Although this approach is fairly robust to identify each vehicle during lane overlap, it limits the applicability to roads with no more than two through lanes.

## 4. Experiment Results and Discussion

The experiment looks to measure dynamic structural responses under normal traffic flow. Several metrics (raw displacement, differential displacement, and frequency response) are to be evaluated and associated with each of the passing vehicles based on its timestamp. The displacement values should not exceed the defined limit at any occasion.

### 4.1. 35-min Displacement Monitoring

**Figure 9:**
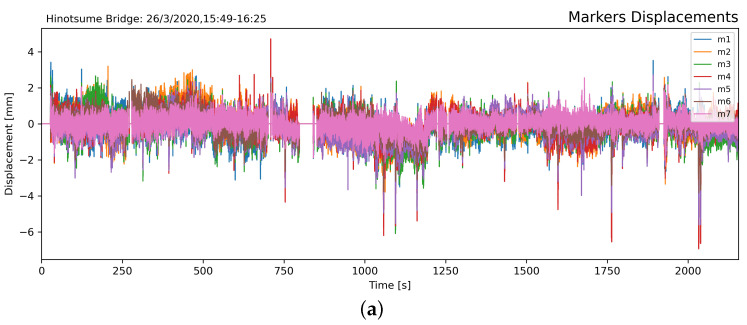

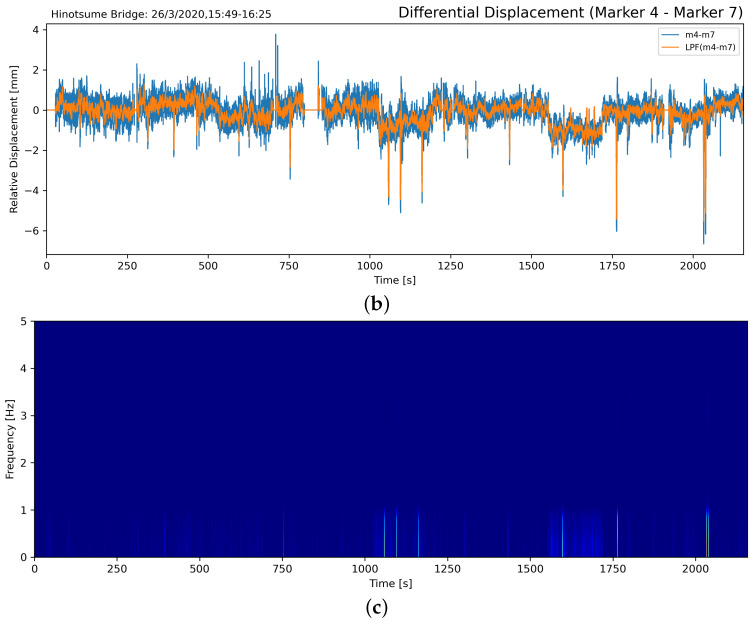
Displacement signal from the seven markers (**a**). Differential relative displacement between M4 (center of the span) and M7 (close to the support pier at the center of the bridge) with a low-frequency band-pass filter (LPF) (**b**). Frequency spectrogram of the differential displacement signal vibration with Hamming window (k = 128) (**c**). Short-duration peaks were observed in the displacement signals and the frequency response during large vehicle passes.

Figure 9 shows the markers’ displacement, derived differential displacement, and frequency spectrogram. The largest displacement during the experiment was 6.944 mm in M4 and still within the limit defined in Table 1 (25 mm, 1000th of the 25 m span). At any occasion during the experiment, neither measured raw displacement (Figure 9a) nor differential displacement (Figure 9b) exceeded the load rating of Hinotsume Bridge. This indicates that no overweight vehicle passed over the bridge and the displacement in the vertical direction had all been within the range of elastic deformation. The constant low-amplitude vibration in the displacement signals could be attributed to sensors’ noises and spatial aliasing in the camera system and DIC process [33].

Passes of large vehicles resulted in large displacement in the markers that are clearly observable from the baseline. The displacement is dominated by first-mode curvature, shown as a single, low-frequency peak in the spectrogram (Figure 9c). A notable low-frequency response (peaks at ≈0.3 Hz) occurred at the same point as its differential displacement signal.

This could be useful to identify the bridge’s structural response to loading forces, which was excited exclusively from large vehicular traffic. However, passes of large vehicles might not always result in large structural displacement as the vehicle could possibly be lightly loaded; the axle load may have been spread further apart; or the possibility of vibration superposition from multiple vehicles/axles, which canceled each other.

The temporary drift observed in the baseline of differential displacement signal, i.e., *t* = 1020–1200 s and *t* = 1650–1800 s, could be attributed due to image disturbance from loose attachment, heat haze, or shadow cast on the markers [37]. Such occurrences simply affect the accuracy of DIC facet position detection rather than imply constant load on the bridge.

### 4.2. Traffic Counting

**Figure 10:**
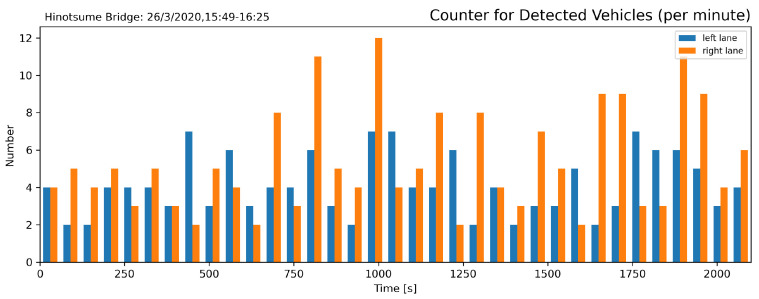
Traffic count in a one-minute segment. Traffic was calm with an average of 4.13 vehicle/min eastbound and 3.25 vehicle/min westbound.

Traffic count during the duration of the experiment is shown in Figure 10. Among the detected 336 vehicles passes, 188 were going eastbound (left-going) and 148 westbound (right-going) with calm traffic flow. The slight difference could be attributed to the fact that the bridge is located on a feeder road to Umaki Interchange (Japanese Expressway E75), situated nearby to the east, which many vehicles head towards to reach other places.

**Figure 11:**
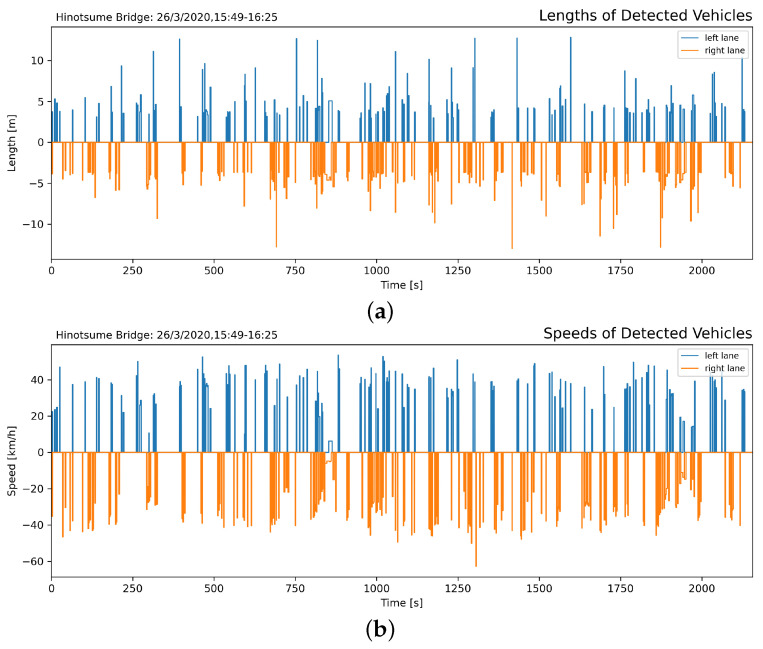
Length of detected vehicles in both lanes on the bridge (**a**). The length was evaluated from the passing vehicles’ pixelwise width in the frame, using different scaling factors for each lane as the left-going is closer to the camera: 36.55 mm/px (left-going) and 38.38 mm/px (right-going). The speed of detected vehicles (**b**) was evaluated from the distance traveled and its duration from a particular ID.

**Figure 12:**
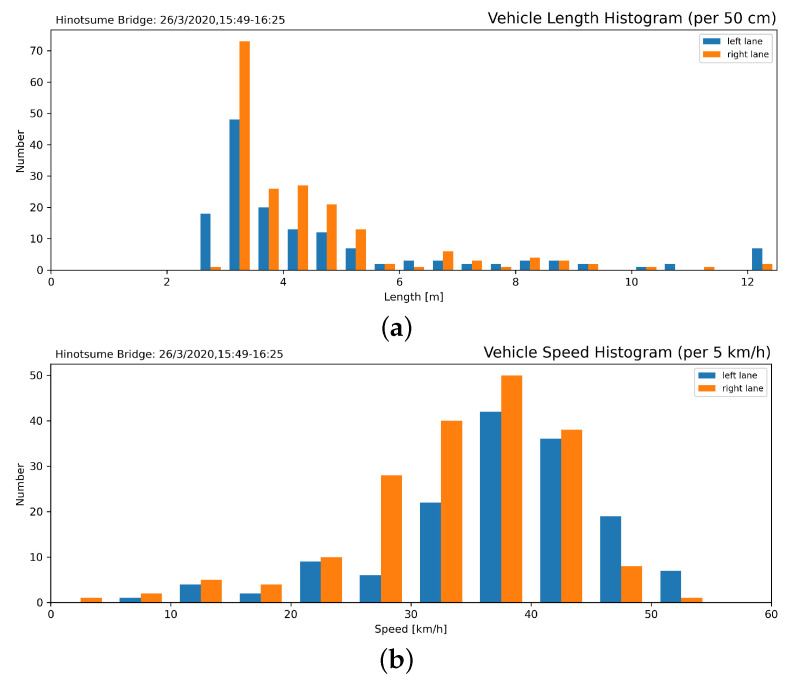
Vehicle length histogram (**a**) shows that Japanese light *kei* cars (<3.4 m) were most common. Passing speed histogram (**b**) shows that drivers adhered to the enforced speed limit (50 km/h).

In addition, vehicle speed and length were evaluated in relation to identify the vehicular loads, shown in Figure 11, further classified in Figure 12. Traffic was dominated by small and compact cars with occasional large trucks. Besides, as the experiment was conducted while the inspectors stood on the shoulder of the road, drivers might voluntarily slow down slightly to ensure the safety of their surroundings.

**Figure 13:**
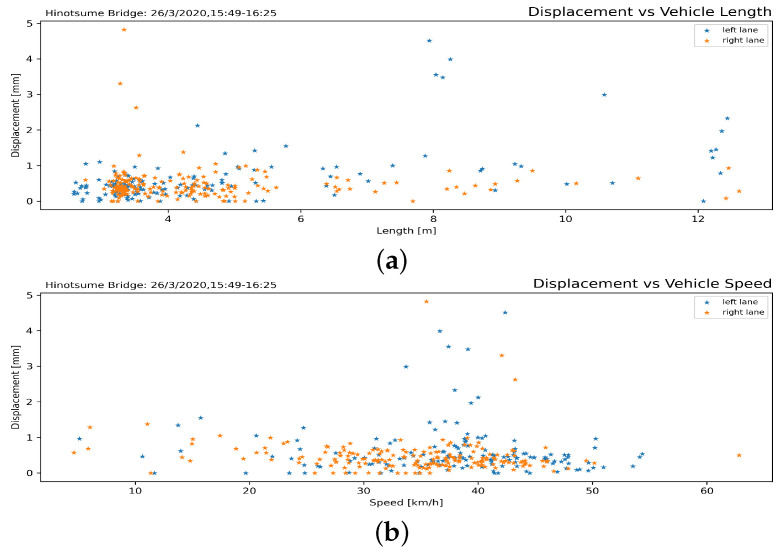
Bridge displacement according to vehicle lengths (**a**) and speeds (**b**). Most passes resulted in displacement less than 1 mm. The vehicle needed to travel at substantial speed to induce substantial structural displacement on the bridge. Several small vehicles were cross-recorded having large displacement due to crossing the bridge at the same time as a large vehicle.

Displacement of markers obtained from DIC analysis, accompanied by its timestamp, was cross-referenced with the timestamp from the traffic counting process to obtain correspondence between the passing vehicles and induced displacement on the bridge. Figure 13 shows the relationship between the markers’ displacement and passing vehicles’ length and speed. Large displacement was only observed during the passes of large vehicles longer than 8 m. As there were no overspeeding large vehicles, the effect of speed in the amplification of force dynamics could not be observed.

### 4.3. Structural Response from Large Vehicle Passes

Bridges will often only exhibit a notable structural displacement response from a sizable vehicular load. AASHTO and FHWA (Federal Highway Administration, US) defined truck configurations and dimensions employed for load test on a bridge for traffic load [61,62], summarized in Table 2. These large vehicle configurations were incorporated for detection in the traffic counting system to determine the bridge’s structural displacement from the traffic load.

Vehicle of interest (large vehicle) was classified based on the estimated vehicle length, given that the weight of the trucks could not be directly measured from the video feed. Although there is no exact specification on vehicle length for bridge live loading in Table 2, AASHTO exemplifies that the standard load trucks’ total length are 3.66 m (12${}^{\prime }$) longer than their outside axle spread [61]. Considering that the shortest specified axle spread in the list is 4.27 m, only detected vehicles longer than 7.9 m (26${}^{\prime }$) were selected for displacement analysis.

**Figure 14:**
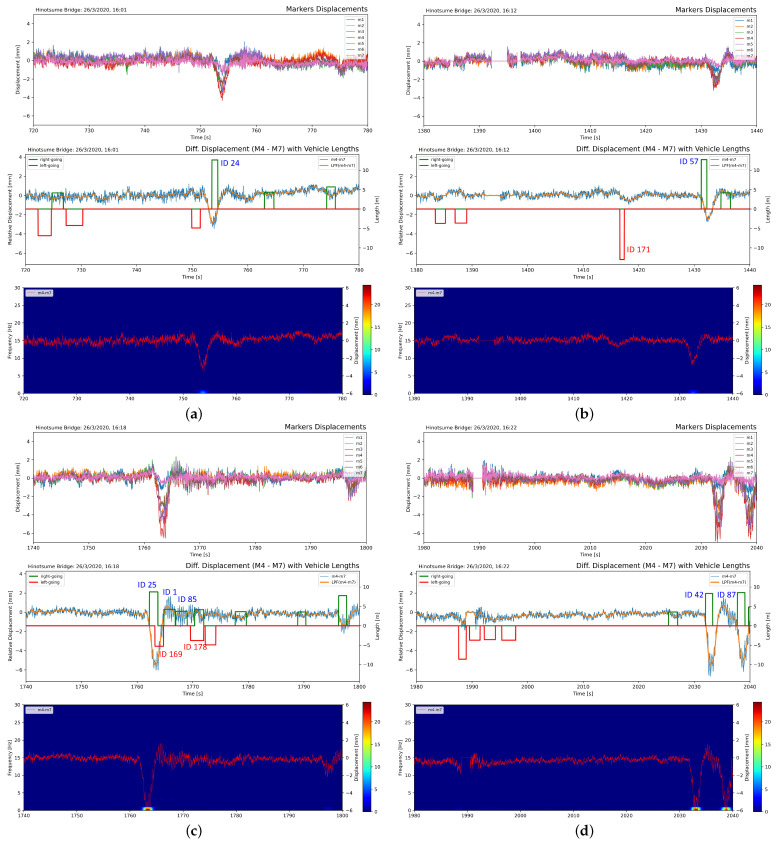
One minute segment of raw marker displacement, differential displacement between M4 and M7, and vibration frequency response at 16:01 (**a**), 16:12 (**b**), 16:18 (**c**), and 16:22 (**d**). The identified passing vehicles in the passes are mentioned in Table 3. Low-frequency vibration spectrum was observed during the passes of large, right-going vehicles while the passes of small vehicles did not result in notable vibration as in the earlier part of (**a**,**b**).

Table 3 shows four occasions of large vehicles’ passes among such passes, which resulted in a differential displacement larger than 2.5 mm throughout the duration of the experiment (Table A1). The corresponding raw displacement signal, differential displacement signal, and frequency response of those passes in a one-minute segment are shown in Figure 14.

The largest peak displacement was consistently observed in M4 (center of the span) with the peak displacement tending to be smaller towards both ends of the span. This agrees with the prior finding that the first-mode curvature constantly comprises the largest component of traffic-induced displacement at all various speeds and weights of the passing vehicles [63].

Consecutive passes of vehicles resulted in sustained vibration, which was initially excited from the pass of a large vehicle. The vibration larger than baseline lingers until the last trailing pass, as shown in Figure 14c, with distinct frequency response peaks observed in Figure 14d despite the fact that its displacement signal has not returned to baseline. Passes of left-going large vehicles resulted only in small displacement compared to right-going ones, shown in Figure 14b. This could be attributed to the fact that the displacement markers were placed closer to the right-going lane and torsional vibration occurs on the span.

**Figure 15:**
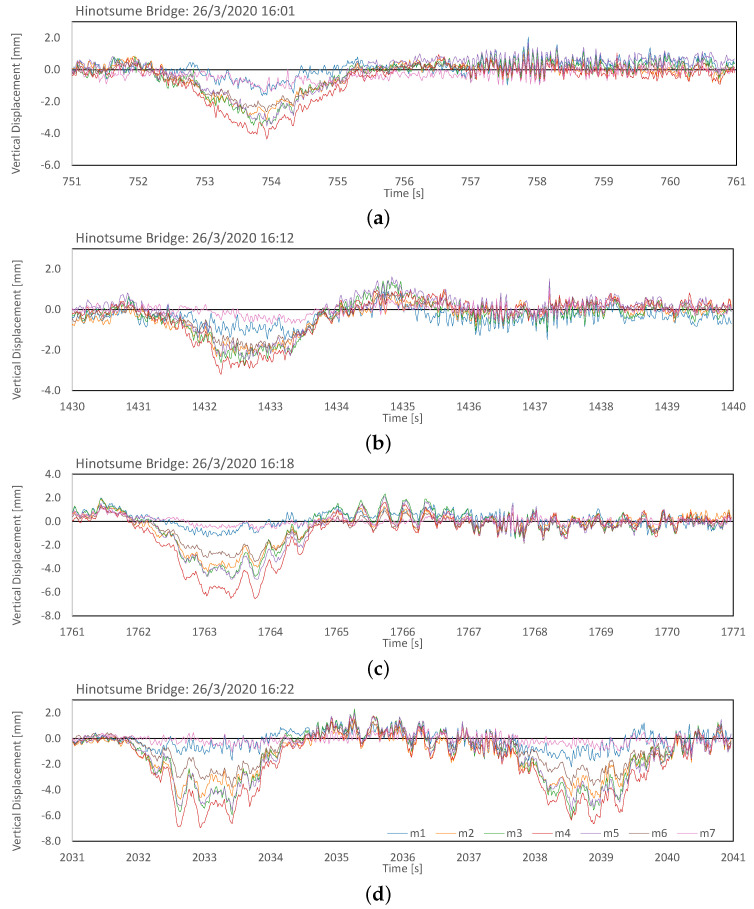
Ten-second segment of marker displacement upon large vehicles passes at 16:01 (**a**), 16:12 (**b**), 16:18 (**c**), and 16:22 (**d**). A notable first-mode vibration component (0.3 Hz) was observed on the markers for the duration of approximately 3 s upon excitation from the pass of a large vehicle, followed by high-frequency small-amplitude transient damped vibration. Consecutive pass(es) of either large (**d**) or small (**c**) vehicles following an initial excitation from a large vehicle’s pass resulted in a sustained, 3-Hz vibration.

**Figure 16:**
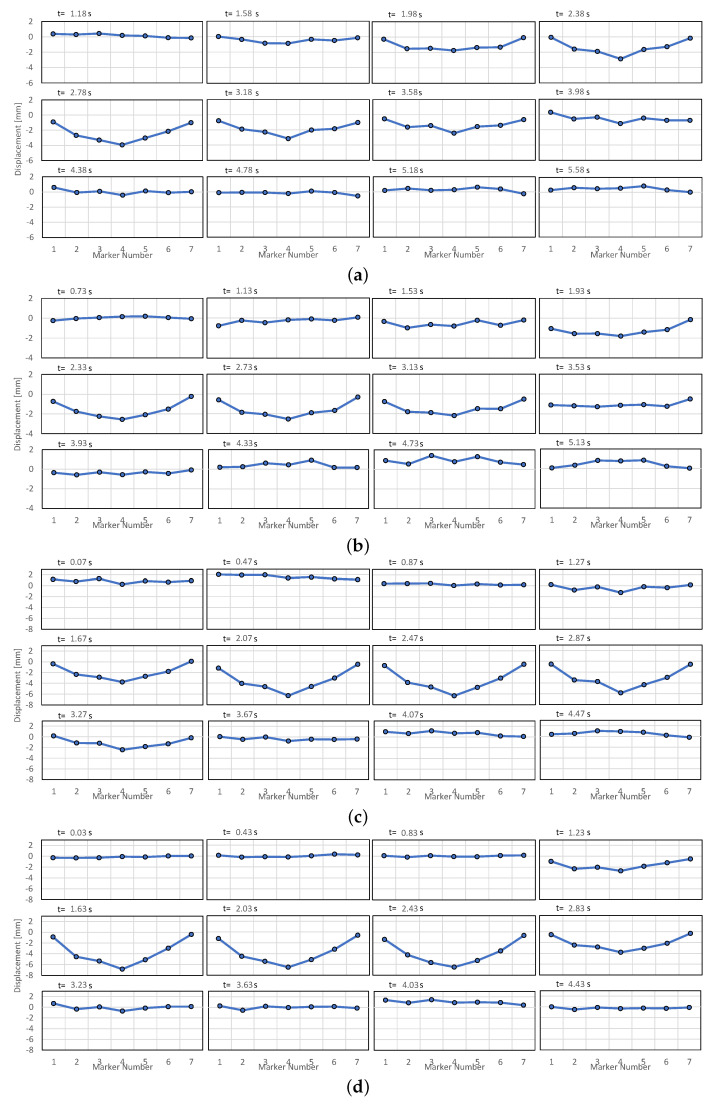
Bridge deck deflection at 16:01 (**a**), 16:12 (**b**), 16:18 (**c**), and 16:22 (**d**) Time offset follows the segment in Figure 15 with a 0.4 s interval between images that start just before the moment of excitation. The deflection shows a downward movement during a large vehicle’s pass with the largest displacement consistently observed towards the center of the bridge span.

The marker displacement signal from the four passes is presented in more detail in Figure 15. All markers started to vibrate rather simultaneously after the initial excitation from the pass of a large vehicle. This could be attributed to the stiff plate-girder construction of the span and the placement of the markers on the guardrail rather than directly on the deck. The corresponding deck deflection of the span during the passes’ duration is illustrated in Figure 16. Damped vibration with first mode at ≈0.3 Hz could be consistently observed after the initial excitation.

## 5. Conclusions

Application of video-based displacement measurement and a traffic counting system was implemented on a plate-girder bridge to measure traffic-induced bridge deck displacement and vibration. Owing to the inability to directly measure vehicles’ weight, the system instead relies on the estimated vehicle length to classify the traffic-induced structural displacement.

Notable displacement on the bridge could be observed from the passes of large vehicles in the lane closer to where the markers were installed, and less so from smaller vehicles and from the opposite lane. Vehicle-induced frequency response could be observed more clearly from consecutive passes of large vehicles in the same lane.

Implementation with simpler image-based displacement measurement methods such as marker centroid tracking or phase correlation could be employed in the future to achieve real-time performance of the system for continuous in situ structural health monitoring.

## Figures and Tables

**Table 1:** Criteria for displacement limit on girder bridge. Compound load type (vehicular and pedestrian) has a stricter displacement limit criteria as pedestrian is more sensitive to movement due to the absence of a direct suspension system.

Bridge Construction	Load Type	Displacement Limit
steel, aluminum, or concrete	vehicular only	span/800
steel, aluminum, or concrete	compound	span/1000
steel, aluminum, or concrete (cantilever)	vehicular only	span/300
steel, aluminum, or concrete (cantilever)	compound	span/325
timber	compound	span/425

**Table 2:** Standard trucks for bridge impact testing. The three-axle truck (*) may have variable outside axle spread up to 13.4 m (44${}^{\prime }$) with the same tractor configuration as the two-axle truck. Longer combination vehicles (**) are not allowed in Japan road networks.

Configuration	Gross Weight (ton)	Outside Axle Spread (m)
Two-axle truck	13.64	7.85
Three-axle truck *	24.55	10.67
Four-axle trailer	29.09	11.13
Five-axle trailer	36.36	20.21
Six-axle trailer	40.91	20.37
Five-axle double trailer	36.36	23.26
Seven-axle double trailer **	54.55	32.41
Eight-axle double trailer **	56.36	27.84
Nine-axle double trailer **	67.27	40.03
Seven-axle triple trailer **	60.00	33.29

**Table 3:** Passing Large Vehicles.

Time (Vehicle ID)	Vehicle	Length (m)	Speed (km/h)
16:01 (24)	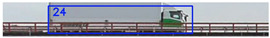	12.27	37.11
16:12 (171 and 57)	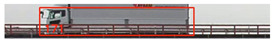 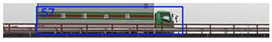	12.62 12.36	43.09 39.38
16:18 (25)	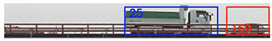	8.26	36.65
16:22 (42 and 87)	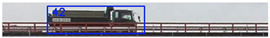 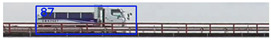	7.94 8.14	42.36 39.11

## Data Availability

The data presented in this study are available on request from the corresponding author. The data are not publicly available due to the large size of the video files and privacy concern regarding the appearance of license plates of the passing vehicles.

## Appendix A

**Table A1:** Passes with large displacement (>2.5 mm). The listed duration represents the duration when differential displacement larger than set 2.5 mm was continuously observed throughout the pass. Small vehicle passes during such duration were being misdetected as having large displacement.

Time (ID)	Pass Duration [s]	Traffic Counting Camera
16:00 (24)	1.12	
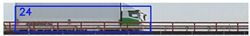
16:06 (20)	1.82	
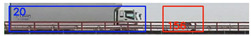
16:07 (61)	1.53	
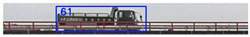
16:08 (81)	1.38	
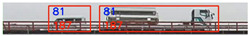
16:15 (13)	1.58	
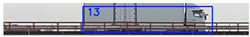
16:18 (25)	1.67	
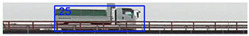
16:22 (42)	1.57	
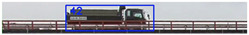
17:23 (87)	1.62	
